# Excretory Function of Intestinal Tract Enhanced in Kidney Impaired Rats Caused by Adenine

**DOI:** 10.1155/2016/2695718

**Published:** 2016-11-15

**Authors:** Yu Yun, Tao Gao, Yue Li, Zhiyi Gao, Jinlian Duan, Hua Yin, Weigang Duan

**Affiliations:** ^1^Kunming Key Laboratory of Molecular Biology for Sinomedicine, Faculty of Basic Medicine, Yunnan University of Traditional Chinese Medicine, Kunming 650500, China; ^2^School of Basic Medicine, Kunming Medical University, Kunming 650500, China

## Abstract

The main aim of the study was to prove the compensative effect of intestine for renal function. Rat kidney was impaired by intragastrically administrating adenine (400 mg per day for 5 days). Intestinal tract was harvested and equally divided into 20 segments except cecum. Kidneys were harvested and histologically examined with hematoxylin-eosin staining kits. Uric acid, urea (BUN), and creatinine in serum were determined with assay kits, and BUN and creatinine in every intestinal segment were also determined. The results showed that adenine was able to increase uric acid level in serum from 20.98 ± 6.98 *μ*g/mL to 40.77 ± 7.52 *μ*g/mL and cause renal function damage with BUN (from 3.87 ± 0.62 mM to 12.33 ± 3.27 mM) and creatinine (from 51.48 ± 6.98 *μ*M to 118.25 ± 28.63 *μ*M) increasing in serum and with abnormally micromorphological changes in kidney. The amount of BUN and creatinine distributed in intestinal tract was positively correlated with those in blood. In impaired renal function rats, the amount of BUN (from 4.26 ± 0.21 *μ*Mole to 10.72 ± 0.55 *μ*Mole) and creatinine (from 681.4 ± 23.3 nMole to 928.7 ± 21.3 nMole) distributed in intestinal tract significantly increased. All the results proved that intestinal tract had excretory function compensative for renal function.

## 1. Introduction

Kidney is an important and vulnerable organ for health. The main function of the organ is to excrete metabolic wastes but easily impaired by many factors including drugs like aminoglycosides [1] and some ingredients transformed from body's own materials like uric acid [2]. Uric acid, as the end-product of purine metabolism in human body transformed by a rate-limiting enzyme, xanthine oxidase/xanthine dehydrogenase (XDH/XO) [3], is predominantly excreted through kidney [4]. Uric acid is almost insoluble in body fluids and will be precipitated in kidney tubules, articular cavities, and other peripherial interstitial spaces if the concentration of uric acid is above normal value (above 70 *μ*g/mL or 420 *μ*M) for a long time [4]. In other animals (apart from primates, some birds, and reptiles), uric acid is able to be transformed to allantoin and other substances through uricase/urate oxidase (UOX) [5], which are more soluble. The precipitation of uric acid and its urate in turn will result in gout and most frequently cause renal dysfunction.

Hyperuricemia is a common factor that damages renal function [2] then decreases the ability to excrete metabolic wastes. As the main wastes, urea (blood urea nitrogen (BUN)) and creatinine in serum are used as the important indexes to diagnose and predict renal function, though not sensitive at early stage [6]. Actually, kidney, though, is the main but not the only organ for excretion. Then, other organs, like skin [7] and intestinal tract [8], could partly undertake the task, because some metabolites could be detected in sweat [7], and intestinal dialysis [9, 10] was helpful for renal failure patients to improve their symptoms by lowering BUN and serum creatinine. However, the role of intestinal tract in excretion was ignored to a large extent in both clinic and experiment studies, and the fact was not proved with experimental results. According to the opinion of animal evolution, intestinal tract developed earlier than kidney and urinary system. The intestinal tract was responsible for both absorption and excretion in animals whose kidney and urinary system were not evolved like in* Caenorhabditis* [11] and* Schistosoma mansoni* [12] or not developed.

Therefore, it can be believed that intestinal tract could still keep some function for excretion, though the evidence was not fully understood. Overload of adenine can cause renal function damage at least partly by increasing blood uric acid [13]. The present study will clarify the relationship of metabolic wastes (BUN and creatinine) between blood and intestinal tract in impaired renal function rats caused by adenine.

## 2. Materials and Methods

### 2.1. Materials

Clean, female, Sprague-Dawley (SD) rats aged 2 months and weighing 180–200 g were obtained from Jianyang Dashuo Science and Technology Ltd., Chengdu, China (certification number SCXK (Chuan) 2008-24). Rats were housed in temperature-controlled (22°C) and humidity-controlled (45–55%) conditions, under natural light. This project was approved by the Experimental Animal Committee of Yunnan University of Traditional Chinese Medicine in China.

Uric acid was produced by Tokyo Into Industrial Co., Ltd. (Tokyo, Japan). Adenine was produced by Shanghai Yuanye Biotech Ltd. (Shanghai, China). Standard lotion of uric acid (1000 *μ*g/mL, 5952 *μ*M), uric acid assay kits of phosphotungstic acid method, creatinine assay kits of picric acid method, and blood urea nitrogen (BUN) assay kits of diacetyl monoxime method were produced by Nanjing Jiancheng Bioengineering Institute (Nanjing, China). Ultrapure water was produced by Milli-Q water purification system manufactured by EMD Millipore Group (Darmstadt, Germany). Other reagents used were of analytical reagent grade and made in China.

The multimicroplate reader of Infinite 200 Pro was manufactured by Tecan Group (Mannedorf, Switzerland). The light microscope was manufactured by Nikon Corporation (Tokyo, Japan). Other instruments used in the present study were made in China.

### 2.2. Animal Treatment

SD rats were treated normally for 3 days and were randomized to 2 groups, namely, normal group and kidney impaired group. Rats in kidney impaired group were intragastrically treated with adenine (400 mg/kg per day) for 5 days, and rats in normal group were served as control and treated with normal saline of the same volume. All the rats were fasted for 36 h before being sacrificed.

When the last administration was taken for 2 h, rats were intraperitoneally anaesthetized with urethane (1 g/kg). The abdomen of rat was opened, blood samples were drawn via the abdominal aorta, and stomach and intestinal tract from duodenum to rectum were harvested. The stomach juice was directly collected. The intestinal tract was equally divided into 20 segments (about 5 cm per segment) according to the length of intestinal tract except cecum (about 10 cm). The intestinal juice in the segments was collected when a segment was cut, the inner wall of the intestinal segment was rinsed with normal saline of 200 *μ*L, and the two parts of liquid sample were combined as intestinal sample for assay.

Kidneys were harvested, perfused with normal saline and then with 10% formaldehyde, and finally fixed in 10% formaldehyde. When the blood sample was coagulated, the serum sample was obtained by spinning at 3,000 ×g for 5 min at 4°C.

### 2.3. Histological Examination

Rat kidneys in each group were immersed in 10% formaldehyde until a routine histological operation was conducted. Paraffin sections of the kidney were cut parasagittally or paracoronally (10 *μ*m). The sections were stained with a hematoxylin-eosin staining kit. Images were obtained with the light microscope.

### 2.4. Uric Acid Assay

The concentration of uric acid (*μ*g/mL) in the serum samples, stomach juice, and intestinal samples was assayed with uric acid assay kits according to the standard operation procedure (SOP) provided by the producer.

### 2.5. BUN Assay and Creatinine Assay

The concentration of BUN (mM) and creatinine in the serum samples, stomach liquids, and intestinal samples was assayed with urea assay kits and creatinine assay kits according to the SOP provided by the producer, respectively. The content of BUN (*μ*Mole) and creatinine (nMole) in the intestinal sample was calculated by multiplying its concentration and volume.

### 2.6. Statistical Analyses

Total BUN or creatinine in rat serum was calculated by multiplying its concentration in serum and 1/26 of its body weight (the total blood is about 1/13 of the body weight, and the serum is about 1/2 of the blood). Total BUN or creatinine in rat intestinal tract was calculated by summing up its content in every intestinal segment together. The IS ratio of BUN or creatinine between intestine and serum was calculated by formula “IS ratio = (total in intestine)/(total in serum).”

Normal saline was used as control. Values were expressed as mean ± SD or mean ± SE. Student's *t*-test was performed to compare means with the normal group. Statistical significance was accepted at *P* < 0.05.

## 3. Results

### 3.1. Impaired Renal Function Caused by Adenine

**Figure 1:**
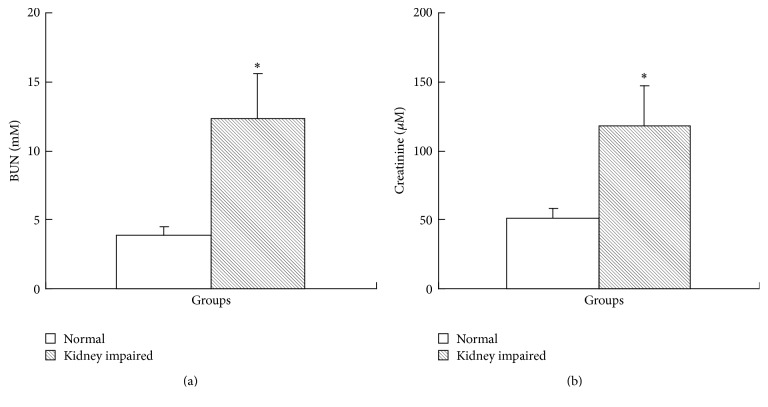
Rat renal function assay (mean ± SD, *n* = 10). Intragastric administration of adenine was able to cause renal function damage. The BUN in kidney impaired group (a) was 12.33 ± 3.27 mM, much higher than that (3.87 ± 0.62 mM) in normal group (*P* < 0.05), and the creatinine in kidney impaired group (b) was 118.25 ± 28.63 *μ*M, also much higher than that (51.48 ± 6.98 *μ*M) in normal group (*P* < 0.05). Rats in kidney impaired group were intragastrically treated with adenine (400 mg/kg × 5 days), while those in normal group were treated with normal saline of the same volume. ^*∗*^  versus normal group, *P* < 0.05, Student's *t*-test.

**Figure 2:**
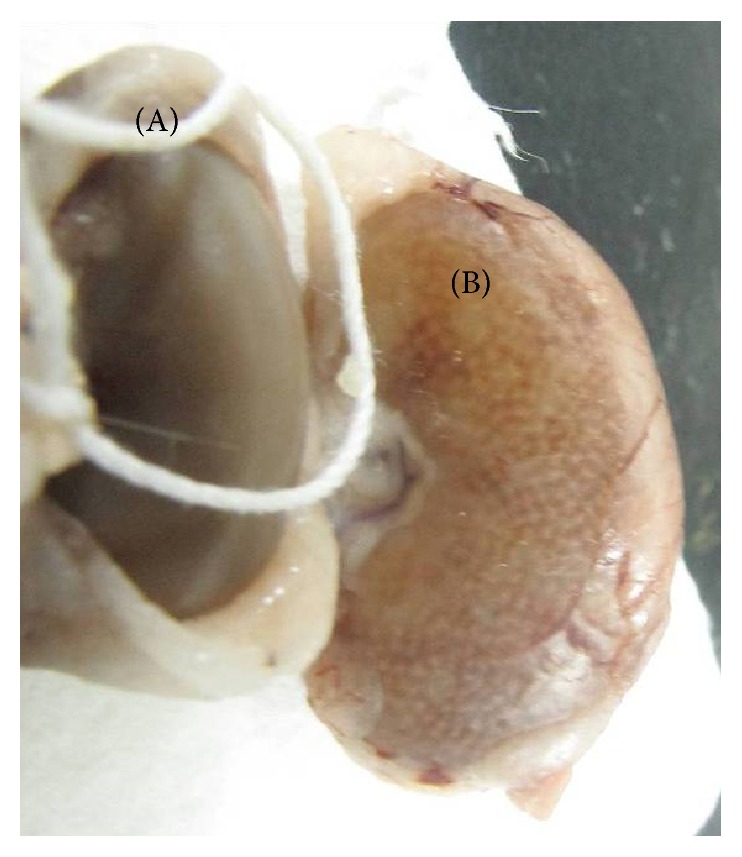
Gross morphology of rat kidney in normal group (A) and kidney impaired group (B). Rats in kidney impaired group were intragastrically treated with adenine (400 mg/kg × 5 days), while those in normal group were treated with normal saline of the same volume. The kidney (A) in normal group was normal, with a smooth surface (weighing 0.935 ± 0.113 g); and the kidney (B) in kidney impaired group was swollen with numerous white dots (weighing 1.917 ± 0.156 g).

The results of renal functional assay showed that, in kidney impaired group, both BUN level (Figure 1(a)) and creatinine level in serum (Figure 1(b)) increased and suggested that the renal function in kidney impaired group was impaired. Kidneys in kidney impaired group were swollen with numerous white dots (Figure 2), much different from those in normal group.

**Figure 3:**
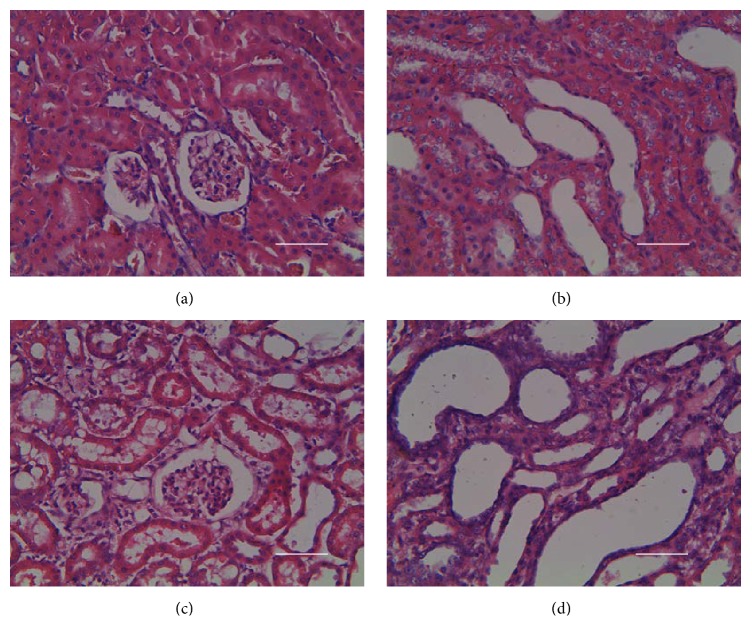
Micromorphology of rat kidneys in normal group ((a) and (b)) and kidney impaired group ((c) and (d)). Nephrocytes in normal group were piled tightly ((a) and (b)); there were no swelling signs in glomeruli (a) and tubules (b). While in kidney impaired group cells in the glomeruli were swollen and the capsular space was dilated (c), the wall of tubules became thin and piled loosely, and the space of tubules was also dilated (d). Rats in kidney impaired group were intragastrically treated with adenine (400 mg/kg × 5 days), while those in normal group were treated with normal saline of the same volume. Bar = 500 *μ*m.

**Figure 4:**
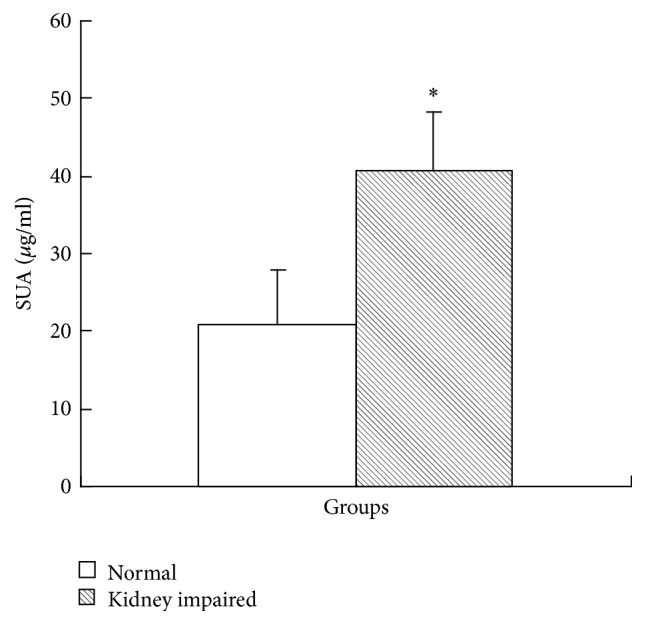
Uric acid in serum in kidney impaired group increased to a higher level (mean ± SD, *n* = 10). The concentration of uric acid in kidney impaired group was 40.77 ± 7.52 *μ*g/mL (242.5 ± 44.73 *μ*M), much higher than that in normal rat serum (21.93 ± 6.98 *μ*g/mL (124.5 ± 41.5 *μ*M), *P* < 0.05). Rats in kidney impaired group were intragastrically treated with adenine (400 mg/kg × 5 days), while those in normal group were treated with normal saline of the same volume. ^*∗*^  versus normal group, *P* < 0.05, Student's *t*-test.

The results of histological examination (Figure 3) showed that the obvious micromorphological damage was caused, though the serum level of uric acid in kidney impaired group was not high enough to diagnose hyperuricemia (Figure 4). In kidney impaired group, cells in the glomeruli were swollen and the capsular space was dilated (Figure 3(c)), the wall of tubules became thin and piled loosely, and the space of tubules was also dilated (Figure 3(d)).

The concentration of uric acid in normal rat serum was 21.93 ± 6.98 *μ*g/mL (124.5 ± 41.5 *μ*M, Figure 4), much lower than the conference value of adult man (149–416 *μ*M). As for rats in kidney impaired group, the concentration of uric acid in their serum was much higher (40.77 ± 7.52 *μ*g/mL) (Figure 4).

### 3.2. BUN in Intestinal Tract

**Figure 5:**
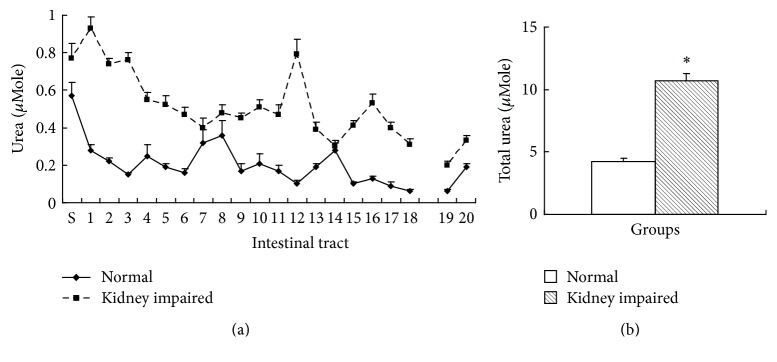
BUN in intestinal tract (mean ± SE, *n* = 10). There were 20 segments of intestinal tract except cecum, and the content of BUN was expressed as *μ*Mole (molar concentration multiplying volume) per segment. BUN in every segment of intestinal tract in kidney impaired group was higher than that in normal group (a), and the total BUN in intestinal tract in kidney impaired group was also higher than that in normal group (b). Rats in kidney impaired group were intragastrically treated with adenine (400 mg/kg × 5 days), while those in normal group were treated with normal saline of the same volume. S, stomach; 1 to 18 at horizontal ordinate meant the 1st to 18th segment of small intestinal tract; 19 and 20 meant 2 segments of colon. ^*∗*^  versus normal group, *P* < 0.05, Student's *t*-test.

BUN is urea, the main metabolic waste from protein catabolism, which is able to diffuse into intestinal tract. There were 20 segments of intestinal tract except cecum, and the content of BUN in every segment and in all the segments was expressed as *μ*Mole. BUN in every segment of intestinal tract in kidney impaired group was higher than that in normal group (Figure 5(a)), and the total BUN in intestinal tract in kidney impaired group was also higher than that in normal group (Figure 5(b)).

### 3.3. Creatinine in Intestinal Tract

**Figure 6:**
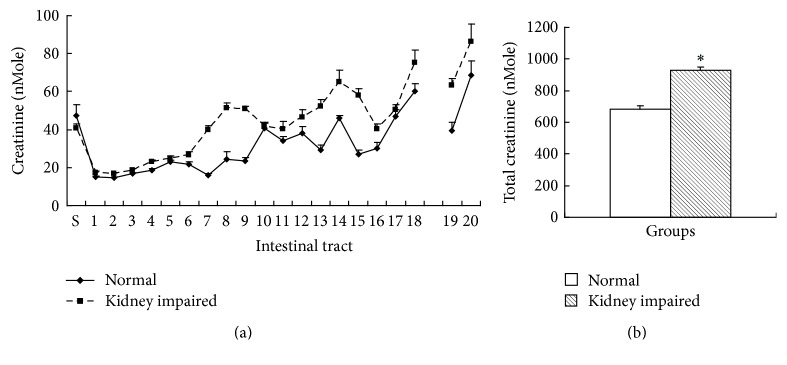
Creatinine in intestinal tract (mean ± SE, *n* = 10). There were 20 segments of intestinal tract except cecum, and the content of creatinine was expressed as nMole (molar concentration multiplying volume) per segment. Creatinine in second and later segment of intestinal tract in kidney impaired group was higher than that in normal group (a), and the total creatinine in intestinal tract in kidney impaired group was also higher than that in normal group (b). Rats in kidney impaired group were intragastrically treated with adenine (400 mg/kg × 5 days), while those in normal group were treated with normal saline of the same volume. S, stomach; 1 to 18 at horizontal ordinate meant the 1st to 18th segment of small intestine; 19 and 20 meant 2 segments of colon. ^*∗*^  versus normal group, *P* < 0.05, Student's *t*-test.

Creatinine is the main metabolic waste from creatine. Creatinine in the second and later segments of intestinal tract in kidney impaired group was higher than that in normal group (Figure 6(a)), and the total creatinine in intestinal tract in kidney impaired group was also higher than that in normal group (Figure 6(b)).

### 3.4. Relationship of Urea and Creatinine between Serum and Intestinal Tract

**Figure 7:**
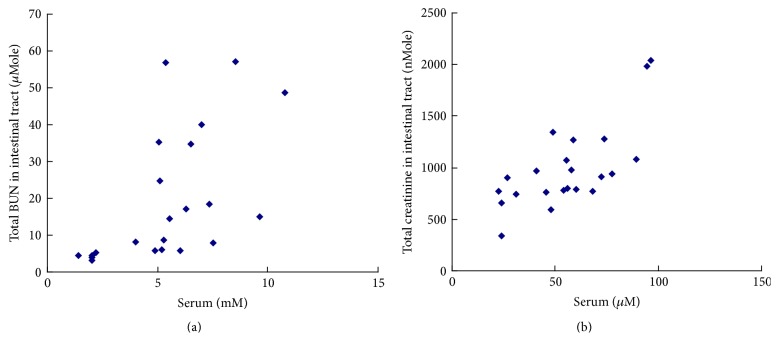
The correlations of BUN (a) and creatinine (b) between serum and intestinal tract (*n* = 20). The relationship in (a) showed that the total BUN (*μ*Mole) in intestinal tract was proportionate to the level of BUN in serum, and the coefficient of correlation (*R*) was 0.582 (*P* < 0.05, Pearson Correlation, 2-tailed). The relationship in (b) showed that the total creatinine (nMole) in intestinal tract was also proportionate to the level of creatinine in serum, and the coefficient of correlation (*R*) was 0.710 (*P* < 0.05, Pearson Correlation, 2-tailed).

The results in Figure 7 showed that the total BUN in intestinal tract was positively correlated with the level of BUN in serum (*R* = 0.582, *P* < 0.05, Figure 7(a)), and the total creatinine in intestinal tract was also positively correlated with the level of creatinine in serum (*R* = 0.710, *P* < 0.05, Figure 7(b)).

## 4. Discussion

Kidney is the most important excretory organ. Generally, intestinal tract is an alimentary organ for digestion and absorption. According to general understanding of evolution, other organs like skin [7] and intestinal tract [8] could also share the excretory function. Although there were clinic clues [9, 10] that the intestinal tract could be an alternative excretory organ, the effect of intestinal tract for excretion was not fully understood. The present study elucidated that metabolic wastes like BUN and creatinine were able to distribute in intestinal tract when rat renal function was normal or impaired.

Adenine is a purine base and is able to transform to 2,8-dihydroxyadenine and uric acid [14]. Both of them are almost insoluble, can precipitate in kidneys (the white dots seen in the kidney in the present study could be the precipitation of them), and then cause renal function damage [14]. Therefore, adenine is a common tool compound to establish animal model of renal failure [15]. The present study found that adenine (400 mg/kg per day for 5 days) was able to result in renal function damage and abnormal micromorphological changes with BUN and creatinine in serum increasing. The results suggested that the mechanism of adenine in inducing renal failure could be associated with the accumulation of both 2,8-dihydroxyadenine and uric acid.

The level of BUN and creatinine in serum was positively correlated with that in intestinal tract. Most metabolic wastes like BUN (urea) and creatinine were theoretically permeable small molecules. In normal group, the level of BUN and creatinine in both serum and intestinal tract was low; and the level increased in kidney impaired group. The results indicated that BUN and creatinine were really able to distribute in intestinal tract. However, the IS ratio of BUN (15.20% ± 1.15%, mean ± SE) was much lower than that of creatinine (182.87% ± 11.2%, mean ± SE). This phenomenon could be caused by microbes whose expressing urease resided in intestinal tract [16], because the distribution trend of BUN in every segment of intestinal tract was consistent to that of microbes [17].

Therefore, when renal function was impaired, the absolute amount of BUN and creatinine increased in both serum and intestinal tract. It can be deduced that if the metabolic wastes distributed in intestinal tract were removed, the wastes in blood could be lowered; and these were supported by a few spotted clinic practices [9, 10]. The excretory effect of intestine could be a compensation for kidney.

Taken together, the present study proved with solid experimental evidence that intestine can compensate renal function in both normal rats and kidney impaired rats.
