# Corrigendum to “Excretory Function of Intestinal Tract Enhanced in Kidney Impaired Rats Caused by Adenine”

**DOI:** 10.1155/2020/8273196

**Published:** 2020-10-19

**Authors:** Yu Yun, Tao Gao, Yue Li, Zhiyi Gao, Jinlian Duan, Hua Yin, Weigang Duan

**Affiliations:** ^1^Kunming Key Laboratory of Molecular Biology for Sinomedicine, Faculty of Basic Medicine, Yunnan University of Traditional Chinese Medicine, Kunming 650500, China; ^2^School of Basic Medicine, Kunming Medical University, Kunming 650500, China

In the article titled “Excretory Function of Intestinal Tract Enhanced in Kidney Impaired Rats Caused by Adenine” [1], there were two errors, which should be corrected as follows:

- The gender of rats used for the experiment was wrongly mentioned as “female.” This should be corrected as “male.”
- In Figure 4 legend, the value of serum uric acid in normal rats “21.93 ± 6.98 *μ*g/ml” should be corrected to “20.93 ± 6.98 *μ*g/ml.”

The authors would like to apologize for any inconvenience caused.
